# Software and Hardware Requirements and Trade-Offs in Operating Systems for Wearables: A Tool to Improve Devices’ Performance

**DOI:** 10.3390/s19081904

**Published:** 2019-04-22

**Authors:** Vicente J. P. Amorim, Mateus C. Silva, Ricardo A. R. Oliveira

**Affiliations:** Department of Computing, Federal University of Ouro Preto, Morro do Cruzeiro Campus, Ouro Preto, CEP 35400-000, Brazil

**Keywords:** wearable devices, operating systems, build tool, performance, prototype, mining

## Abstract

Wearable device requirements currently vary from soft to hard real-time constraints. Frequently, hardware improvements are a way to speed-up the global performance of a solution. However, changing some parts or the whole hardware may increase device complexity, raising the costs and leading to development delays of products or research prototypes. This paper focuses on software improvements, presenting a tool designed to create different versions of operating systems (OSs) fitting the specifications of wearable devices projects. Authors have developed a software tool allowing the end-user to craft a new OS in just a few steps. In order to validate the generated OS, an original wearable prototype for mining environments is outlined. Resulting data presented here allows for measuring the actual impact an OS has in different variables of a solution. Finally, the analysis also allows for evaluating the performance impact associated with each hardware part. Results suggest the viability of using the proposed solution when searching for performance improvements on wearables.

## 1. Introduction

Recently, wearable devices have become a trending technology in the consumer electronics segment. Advances in hardware miniaturization have enabled improvements in wearable products, making them more attractive to final consumers. Devices on the shelves are now unobtrusive, versatile and easy to use, sometimes resembling parts of ordinary clothes. This fact has increased their adoption, assisting individuals in daily tasks and appointments. Different areas embraced a significant number of wearable solutions, from sports [1,2] and entertainment [3,4] to rehabilitation [5,6,7] and general health-care [8,9,10]. Simple and complex prototypes are proposed using specific hardware modules, such as inertial measurement unit (IMU) to recognize human daily activities [11,12,13,14] and falls [15,16,17], heart rate and breath sensors for energy expenditure estimation [18], piezoelectric sensors for energy harvesting [19] and others [20,21,22,23].

Wearable’s applicability areas allow the proposition of an increasing number of novel solutions. As a consequence, the resulting device’s variety also reflects on their requirements and constraints. They can vary from sensor nodes, which have minimum processing requirements without any connectivity [15,24], to glasses including heavy graphical processing and demand for input/output (I/O). Some solutions tend to claim fewer resources, while other ones frequently use more powerful assets. For instance, some devices do not require high processing, and others may use system on chips (SoCs) and specific graphics processing units (GPUs) [4,25].

Verifying a disparity between hardware and software contexts is possible. Wearables performance frequently increases through the inclusion of more powerful hardware components. Additionally, because of the number of software components and their complexity, this scenario gets worse when involving devices making use of OSs. It is common to find the employment of generic solutions, such as Android Wear [26] and Samsung Tizen [27], which are both adapted versions of the software running in smartphones, TVs and other devices. Still, there are other OS options feasible to be applied on wearables context [28,29,30]. Real-time operating systems (RTOS) and regular ones can be adapted to be used within the desired context, including solutions in other applications. Another relevant point is related to the wearable number of hardware components, which may force the use of a more elaborated OS, possibly providing better management of resources.

In previous research [31,32,33], authors conducted an effort to evaluate the performance of common OSs used in wearables. A proper categorization was used as a means to segment and evaluate interesting and relevant features. Results have shown the impact software has in devices specific points and overall performance, presenting a significant discrepancy between better and worst cases.

OSs have a significant performance impact (positive or negative) when used in a wearable. A properly configured software can be around 50% more performance efficient than a conventional embedded OS [32]. If the OSs are well customized and configured, the wearable device can achieve substantial earnings without any hardware change. However, the creation or customization of a specific OS requires significant development effort. This task is, sometimes, even more challenging in the academic field, where researchers frequently produce various prototypes during the life cycle of research.

The work presented in this paper takes OS performance problem into account within the wearable context. Generic solutions often present poor performance, sometimes showing a consistent discrepancy according to the analyzed variable [32]. Additionally, creating a specific OS for each wearable may not be feasible all the time. Due to its complexity, it may require long development time and high-level technical knowledge. Such a scenario becomes even worse when considering the sensors commonly found on wearable equipment. These components may increase the final solution complexity, since it is necessary to port all the legacy hardware to new OS.

### Contributions

In this paper, authors present a tool called wearable create OS tool (WeCOST), that aims to support the development and customization of OSs for the wearable computing context. This tool allows the design of a wearable OS through the specification of functional and non-functional requirements, with the inclusion of software modules to carry out most common hardware sensors and actuators. WeCOST creates an OS version with performance increase using system characteristics as input. Briefly, the main advantages of this tool can be summarized as:

- Reduced time to develop and build custom OSs for wearable solutions;
- Decreased time to support sensors, actuators and other hardware components. Such modules may already be contemplated within WeCOST;
- Software reuse, once the same base can be reapplied to more than one wearable. This point can also cut down development efforts, allowing more time to be used crafting application-level functionalities; and
- Software efficiency can lessen hardware requirements, possibly diminishing associated costs;

The remaining contributions of this paper are:

- A study over the main requirements (functional/non-functional) that commonly affects wearable devices performance;
- A systematic classification of wearable proposals according to specific contexts;
- A functional tool to design, create and customize OSs that fits wearable devices requirements;
- An ubiquitous wearable device prototype that retrieves and process user bio-data in surface mining environments;
- A report including the impact an OS has in different variables performance of a true wearable solution.

The remainder of this article is divided as follows. Section 2 outlines the basic concepts and the necessary background to understand this research. Additionally, this section also presents an assortment of ways to classify current wearables, showing how heterogeneous this environment scope is. Section 3 presents a set of relevant papers associated with the subject covered here. Section 4 shows the main object of this research: a tool to create and customize specific OSs for wearable solutions. This section also presents an architecture description and the main features covered by the tool. Section 5 introduces the prototype created to validate the WeCOST tool, describing its applicability and main functionalities. This section also shows the mining environment where the prototype is inserted. Finally, the measured variables and methodology employed to evaluate the prototype general performance are presented at the end. Section 6 shows the main findings and a discussion about them. Section 7 presents general conclusions, WeCOST limitations and further steps in this research.

## 2. Wearable Appliances

This section presents the necessary background to understand current research. The first part introduces wearables historical evolution, depicting how these devices evolved from simple to complex solutions. The second part presents a comprehensive classification of wearables based on a set of variables and appliances: body location, sensors, applicability, research focus, functional requirements, and connectivity. These ways to classify wearables are particularly important, once they may justify how complex and varied is the environment addressed in this paper.

### 2.1. Historical Evolution

Although currently widely publicized, first wearable electronic devices date back to the late 1960s with the proposition of head-mounted displays (HMDs) [34]. From this period onwards, substantial improvements in concepts, definitions, designs, prototypes, and wearable products occurred, demonstrating how this research field grew [35,36,37]. This area evolved from simple devices to solutions that ubiquitously integrates users’ daily activities, such as vests, glasses, watches, heart-rate monitors, fitness trackers and so on.

Today, this class of devices reached a significant complexity, forcing the review of previous definitions and classifications. Current wearables can be simple, including only sensors and raw data capture. However, in both academy circles and the market, there are already complex solutions that use artificial intelligence algorithms and real-time image digital processing techniques, increasing end-user context awareness with the environment he/she is inserted.

To correctly perceive current wearable’s scope and coverage, classifications can be made from different perspectives. Such categorization helps to understand next steps, besides exposing requirements heterogeneity. Discerning existing wearable types and demands also helps the research presented here. An analysis of wearable’s history and current scenario allows concluding the importance of having a coupled set of hardware and software. The excessive use of computational resources may cause, on both parts (software/hardware), wastage of time and money, consequently increasing research/product costs and delivery time.

### 2.2. Wearable’s Classification

Wearables are currently employed to solve problems in various contexts. Each of these solutions differs in complexity, requirements, applicability, among other variables. Such heterogeneity allows classifying wearables using several aspects. The following classes are some of the most relevant ways to group existing proposals using common aspects, enabling us to quantify the environment complexity and note that any standardization may become a challenging task.

The classification presented in this section may support to perceive devices’ requirements and scope. Despite that fact, classes presented in this research are not meant to be an exhaustive list of all existing solutions. Following subsections classify wearable devices according to the body location where it is used, sensor modules, device applicability area, research-related focus, functional requirements, and network/connectivity.

#### 2.2.1. Body Location

**Figure 1:**
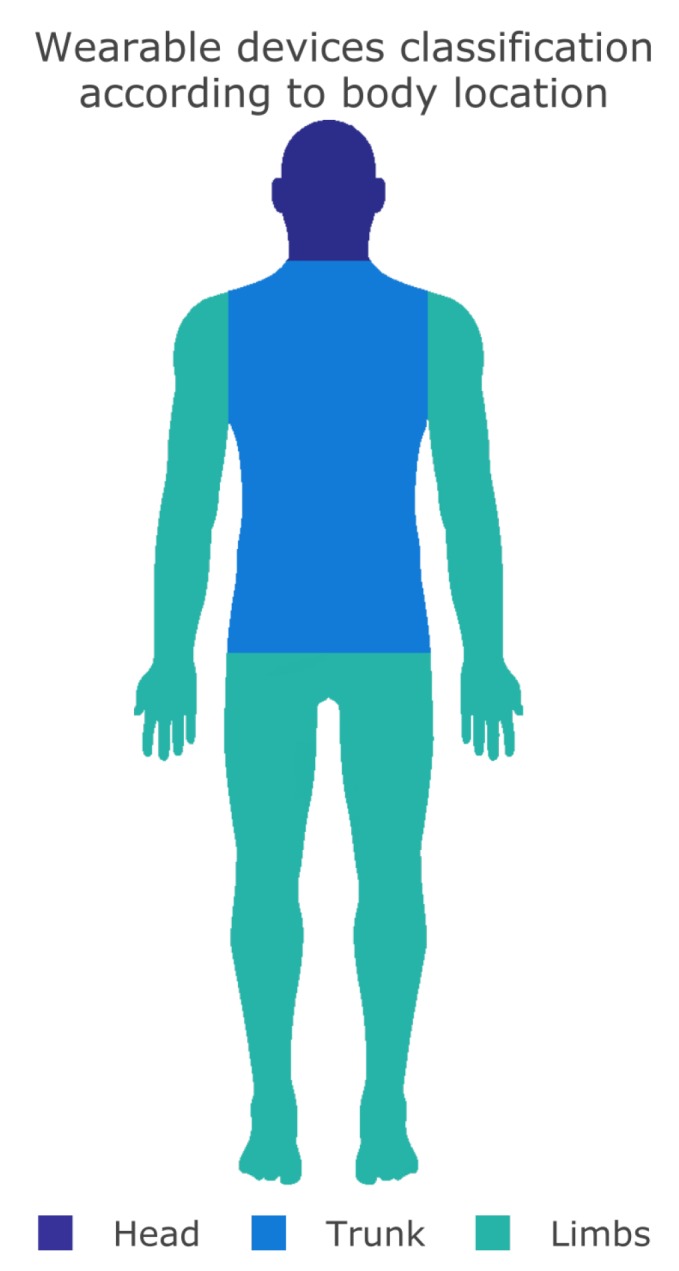
Possible locations where a device can be attached to the human body.

The simplest method to classify wearable equipment is through the place where it is attached to the wearer’s body [38]. Devices’ in-body location can tell a lot about the tasks being performed (wrist, neck, head, waist, legs, an so on). However, a general and more comprehensive classification, using the head, trunk, and limbs division, may provide an overview of wearable type and common locations where these equipment are attached. There are even cases that a wearable can cover two or more body parts, such as a vest. Figure 1 depicts a graphical view regarding the possible locations to attach a wearable in the human body.

Additionally, the list below shows wearable garments organization according to the body location in which they are worn:

- Head: wearable headwear solutions, such as helmets, headphones, hats, HMDs, and so on;
- Trunk: devices attached to the user’s trunk: belts, shirts, necklaces, shoulder-worn devices, and so on;
- Limbs: vests usually connected to body limbs (arms and legs): wristbands, finger-worn devices, some types of jewels, watches, and so on.

#### 2.2.2. Sensors

A wearable solution often senses data from the user and the medium in which it is inserted. Data retrieval is carried out through specific sensors that can provide information according to its nature. In this manner, health data, location, orientation, among others can be obtained instantaneously, and processed locally or sent to a remote server. Then, the number of sensors and their nature may also help to characterize the wearable equipment, acting as a new means to categorize them. Table 1 show a set of sensors frequently used by wearables, as well as their respective role.

**Figure 2:**
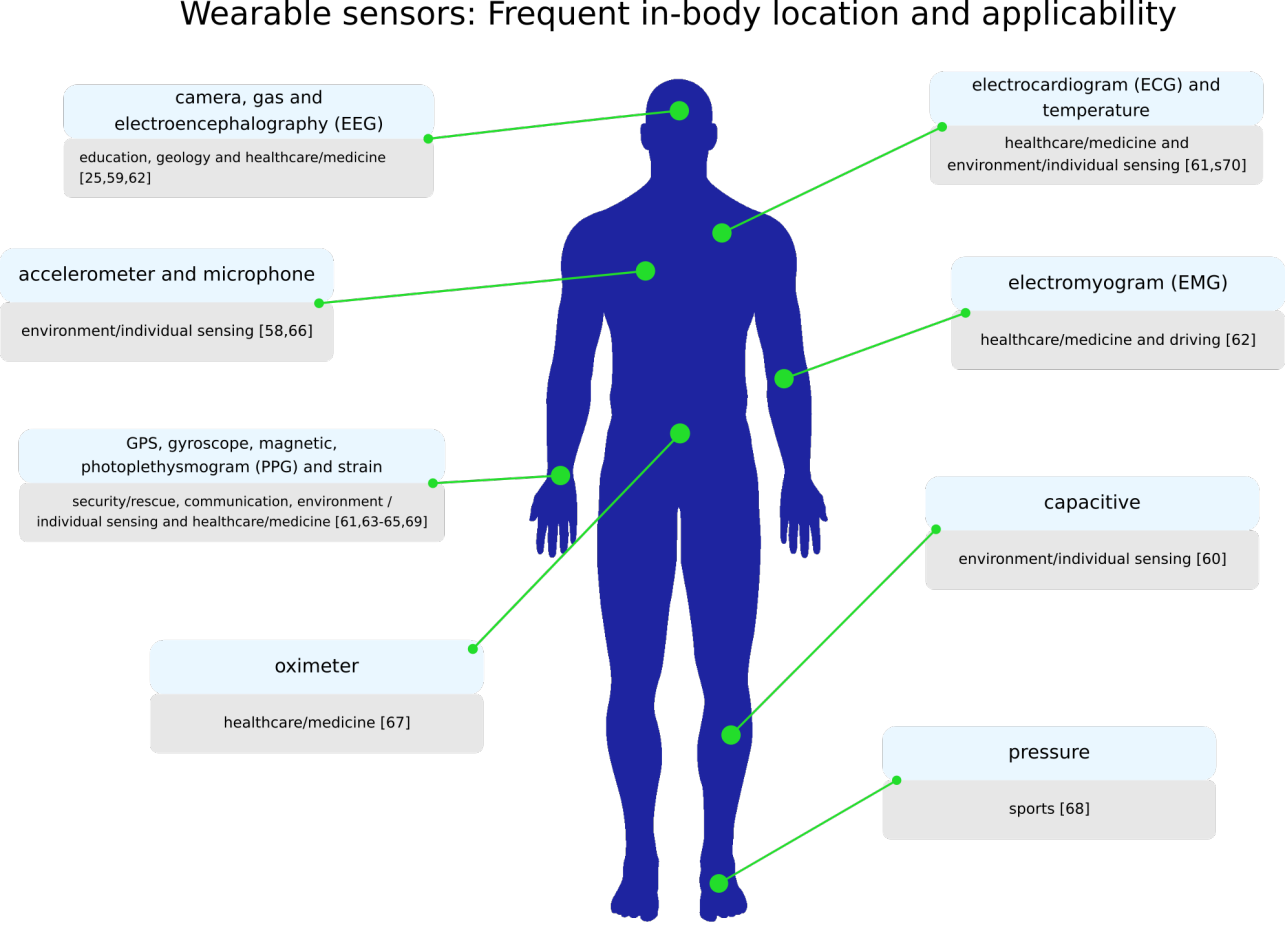
Applicability and common locations where wearable sensors can be positioned. Highlighted points are a few examples of sensors usage. The same hardware can be located in different body parts according to the needs.

Figure 2 associates some popular wearable sensors with the most common sites of their use in the human body. This figure also makes the correlation with applicability areas (Section 2.2.3) in which such sensors can be used. Although extensive, the list presented here can still be increased, with the addition of other less common hardware, such as thermopile sensors, glucose sensors, ultraviolet (UV) sensors, and so on.

#### 2.2.3. Applicability

Wearable computing research evolution brings new application niches. Previously proposed solutions, which were often limited to healthcare/medicine and entertainment, are now applied to a wide variety of contexts. Table 2 presents some of the application areas where wearable devices are currently being used. It is valid to note that, applicability areas comprehensiveness allows having the same wearable being listed in two or more areas.

#### 2.2.4. Research Focus

Wearable devices may be the resulting product of research, especially when taking academic circles into account. Investigation focus indirectly indicates the resulting solution applicability. Thus, another way to classify the wearables involves the research focus associated with each of them. Table 3 provides a comprehensive survey of common research topics found in the current literature. These topics discuss specific problems taking into account wearable computing research.

#### 2.2.5. Functional Requirements

Wearable devices can also be categorized according to their level of complexity and functional/non-functional requirements. The simplest ones do not have a display, primarily working like sensor nodes and being closer to the internet of things (IoT) concept. More elaborate devices can use colored displays and dedicated GPUs. Briefly, wearable devices can also be sorted into the following layers [32]:

- Layer 0: wearables without a display and with focus on data gathering and transmission (e.g., bio-data monitors);
- Layer 1: devices that use a simple display to present text and 2D graphics;
- Layer 2: wearables that provides interaction to the features on Layer 1;
- Layer 3: solutions with 3D-rendering capabilities used in augmented reality/virtual reality (AR/VR) solutions; and
- Layer 4: devices using artificial intelligence (AI) techniques to recognize real world objects. These solutions may use a GPU to speed-up specific tasks.

#### 2.2.6. Network Type and Connectivity

Nowadays, a relevant number of current wearables have some network connection types. These connections permit data to be received or sent, reducing latency to access vital information. When in use, besides the creation of a wearable body area network (WBAN), the network interface may also provide user-private data as input for a remote server. Considering particular wearables, the existence of a communication channel with external entities enables the user to receive help whenever necessary, increasingly approaching it to real-time interaction.

Then, because of its popularity, another possible way to classify the wearables is through the network connection type they use: Ant+, Bluetooth, cellular networks (3G, 4G, 5G, GPRS, GSM, CDMA, TDMA), near field communication (NFC), radio-frequency identification (RFID), Wi-Fi, XBee, Zigbee, and none.

### 2.3. General Requirements

As stated by authors in previous research [31,32], wearables currently vary from simple to sophisticated solutions, that may embed or not, a significant number of internal hardware components. Despite the already existent wearables classification for different contexts [23,38,97], software impact on these devices should be examined taking into account their requirements. Then, performance may be impacted more or less according to the devices’ needs. Solutions with a large number of components tend to claim more resources, while the simple ones may have their needs fulfilled demanding less from hardware.

This paper presents below main variables taken into account when studying OSs impact in wearables general performance.

#### 2.3.1. Processing

The processing load is perhaps one of the most critical variables to consider. A large workload may have multiple impacts on the system as a whole. For instance, the energy expenditure may increase in addition to some tasks delay. Conversely, a more efficient OS can better manage tasks and system resources, avoiding to overload the central processing unit (CPU).

Finally, when analyzing the hardware side, better resources management can even reduce hardware specifications, what in the end will possibly impact the financial costs.

#### 2.3.2. Input/Output

The more requirements a wearable device has, the more sensors/actuators it tends to use. Additionally, network communication with external entities generates a significant mass of data to be received/processed and/or sent by the device. Then, I/O performance can interfere in system requirements fulfilment or even its correctness when breaking time limits on real-time solutions. Each wearable appliance may be affected in some way by I/O performance, according to their needs and internal components.

For instance, a particular wearable used to detect elderly falls must retrieve data at a high enough frequency to calculate whether the user has fallen or not. The failure to meet minimum I/O throughput and processing may lead to incorrect data interpretation, delaying users’ fall notification or even letting to conclude that he did not fell.

#### 2.3.3. Energy Consumption

The energy source limits actual wearable’s autonomy. As soon as the equipment consumes energy, the user must plug back the device to a power source to recharge its batteries. This fact may limit the solution’s up-time.

An OS that spends most of the time processing heavy loads of data tends to require more energy. In the same way, if a significant amount of time is consumed communicating with sensors and other peripherals (especially those with radios, such as GPS, Wi-Fi, and so on), it will also quickly consume available energy. Such impact can even be extended to the wearable costs, as more efficient batteries or hardware may be required.

#### 2.3.4. Availability

Availability represents total time in which the device is active. A wearable is available when it is acquiring and processing data. Eventually, a process may have strong availability requirements depending on the device class. End-users expect that a wearable fits its function as soon as they wear it. Then, availability is even more crucial when involving solutions performing real-time user monitoring. For instance, a device monitoring falls must start processing information as soon as the user wears it. Otherwise, the wearable may lose some data and possibly, a fall will not be detected.

Within the context of OSs for wearables, the authors consider “boot time” as one of the most important variables that may (or may not) ensure a reasonable level of availability. The longer the boot time takes, the more data will be lost.

#### 2.3.5. Digital Signal Processing

Currently, a relevant number of wearables make use of digital signal processing (DSP) techniques to extract raw data from sensors. Especially in the healthcare and personal sensing contexts, such techniques are employed to post-process information acquired by sensors such as ECG, EEG, EMG, PPG, IMUs and so on, removing noise, treating artifacts, applying different filters and extracting features [98]. The use of this post-processing approaches often requires a pipeline of specific operations, which may demand a high processing load [99,100]. Still, the amount of data generated by such sensors can further increase wearable system processing requirements, especially CPU time. This scenario can be worsened when real-time restrictions apply, depending on the wearable product/prototype final applicability.

When the final application is very specific, it is common to have a specialized hardware being used to support DSP-dependent applications. In this case, the hardware project can be synthesized in a real platform aiming to increase general performance [101].

In the absence of a dedicated hardware component to perform DSP operations, the CPU will be shared with other application and system tasks [102]. In such cases, an inefficient OS coupled with a significant processing demand may force the exchange of current hardware for a more advanced one. However, instead of changing the hardware, software improvements can be taken into consideration. An improved OS can better manage system resources, leaving enough room to process DSP operations.

For these reasons, the authors consider that digital signal processing operations must be taken into account when analyzing the performance of wearable OSs.

## 3. Related Work

This section shows related work that has a certain similarity level with the current one. Due to the work novelty, other tools that perform the same functionalities as WeCOST could not be found during the conducted literature review. Then, similar proposals were considered as a means to analyze WeCOST functionalities and performance.

Articles mentioned here focus on four different approaches: OSs performance improvement, existing tools to build OSs, performance impact of OSs, and wearable solutions for mining environments. These four analysis aims to discuss all the relevant contributions attended in the research presented here.

### 3.1. OS Performance Improvement

In the literature, research focusing on operating systems performance improvements are not new. The research presented in [103] aims to speed-up OS performance through changes in compilation stages. However, once this research is relatively old, most of the presented approaches are currently being used in actual OSs.

In recent years, the research area focus has been changed from OSs overall performance improvements to more limited scopes. Research presented in [104] describes a set of memory optimization techniques for multithread OSs. Despite the results shown in the manuscript, this solution has its target in a wireless sensor nodes context.

Other proposals addressing OSs internals can also be found in the literature. In [105], the authors propose a factored operating system targeting manycore platforms. The main idea is to prioritize scalability as a way to take advantage of multicore systems. Advances presented in the article can be used together with the tool proposed in this paper. However, the improvements cannot be used by non-multicore platforms, making it impossible to be applied in simple wearable platforms, such as the one taken into account by the prototype described in Section 5.3.

Related research presented here does not focus on wearable computing systems, which has specific requirements and restrictions. This fact helps do support the use of the tool proposed in this article.

### 3.2. Tools to Build Operating Systems

The work described in [106] presents a tool with a graphical interface that aims to assist users when choosing an OS. Based on the functional requirements listed by the user, the tool can suggest which OS is closer to his needs. Although using similar concepts to those proposed in this research, the tool presented by these authors is pretty limited. After inputting requirement data, only OSs suggestions are made at the end, so that no system is built. Additionally, the given approach is very generic, with no mentions to wearable computing context. On the other hand, the WeCOST tool analyzes functional requirements information for wearables and generates, as output, a usable OS that fits the device needs.

Another relevant tool for embedded OSs creation is Yocto [107]. The Yocto Project is an open-source initiative which takes care of tool maintenance. Aiming the creation of specific-tailored OSs, called recipes, this tool has its focus on producing a Linux distribution for any embedded environment, allowing developers to craft a solution according to their needs. In addition to being famous, with a large and active user base, Yocto is a powerful tool that supports a large number of architectures and software components. Such variety also makes this tool complex, which can be a deterrent for beginning users. Besides that, the lack of a specific bias for wearable computing causes some effort to be put into building and configuring a recipe for such an environment.

Aiming to create embedded Linux OS, the Buildroot tool [108] can also be addressed in the same class of WeCOST. Indeed, this tool also has similarities with Yocto, with a large and significant community of collaborators. In the same way as Yocto, the creation of an initial solution may require a significant development effort, since the tool is a way generic and comprehensive. Although this fact, Buildroot is internally used in WeCOST to manage existing packages as well as the OS initial configuration. In the research presented in this paper, WeCOST holds wearables-specific configurations, while Buildroot is taken into account to provides a bare metal Linux used as a reference OS.

Finally, an embedded OS creation tool called LinuxLink is presented in [109]. This tool has a web interface and works similarly to WeCOST, Yocto, and Buildroot. Although LinuxLink supports a reasonable number of boards and software packages, it does not allow to specify or generate a wearable OS from its general requirements. In fact, despite LinuxLink allows the creation of a large number of OSs, the system has no modules or parts focusing on wearables, as supported in WeCOST.

In general, proposals cataloged in this section has a generic approach, making the development process tough, besides taking more time to complete. In contrast, WeCOST offers the possibility to create specific OSs for wearable products and prototypes. Such specificity eases the development process, making it quick and producing better results, as can be seen in Section 6.

### 3.3. Impact of OSs in Wearables Performance

Embedded systems performance can be upgraded through the addition of new or most advanced hardware. Improvements are also possible through software changes, sometimes reaching the same or even better results. These software improvements are frequently a laborious task, demanding time and development efforts. By its turn, when involving wearables using an OS, performance improvements are possible in different ways, taking into account the final solution/prototype requirements and applicability scenarios. This section presents the articles that are closer to the subject addressed in this research.

Research in [110] shows the development process of a low power RTOS for wearable-specific applications. The paper presents good results regarding memory footprint and energy consumption. However, to fit today’s needs, this OS must take into account another set of constraints. Moreover, the current wearables evolved significantly, in a way they now require more processing power, which may impact other metrics. Indeed, when comparing eRTOS with the tool presented here, the former one lacks flexibility. Each new wearable solution/prototype also requires the development of a new OS version.

Recently, the literature presents no research focusing on wearables OSs performance improvement. However, there is a consistent number of papers depicting the impact an OS has in wearable system general performance. The work presented in [111] tears down and makes a complete analysis of Android Wear OS. Variables such as energy consumption, power, and CPU usage were measured aiming to understand OS efficiency. In the end, the paper lists a set of inefficiencies and their causes, supporting the motivation presented here, and making clear the necessity to increase software efficiency in this area.

The authors already investigated OSs performance impacts on wearable devices. Previous work [31,32,33] measure and compare different variables as a means to figure out the relevance of a well-tuned OS. Results pointed out the need to provide more focus on the wearables software part. In the end, previous research triggered the development of work presented here, once it motivates the creation of a tool to reduce development efforts while maintaining general system performance.

### 3.4. Wearables for Mining

The use of wearables in mining is not a recent topic. As in other areas, this category uses a set of sensors commonly found in wearables of other contexts [23]. The primary challenge here is to build useful and unobtrusive devices, supporting users in daily tasks.

The mining process has different challenges. Toxic gases can be a significant threat to underground mine workers. In surface mines, prolonged exposure to the sun, humidity extremes, and others can cause discomfort or even more severe health problems. Despite the existence of many solutions on the commercial area [112,113,114], this section focuses only on recent research applied to surface mining.

In [115], the authors mention a wearable device for coal miners. A transmitter and a receiver unit constitute this system. The transmitter unit contains an Arduino Mega board controlling a set of components such as sensors, liquid crystal displays (LCD), wireless transceiver modules called NRF, and RFID radios. The receiver unit consists of an Arduino Uno board connected to the NRF module and LCD. The transmitter monitors the sensors and sends the resulting information to the receiver.

Further actions are demanded when sensors detect abnormal values. This solution has almost the same applicability scenario as the wearable presented here. The paper does not conclusively shows a wearable solution. Although the authors reveal some architecture details, the pictures only depict tests using a circuit.

A wearable solution presented in [116] shows a smart helmet used by coal miners. The system monitors toxic gas levels looking for elements with a higher concentration than pre-established limits. The paper outlines a system block diagram and the mechanical helmet project. Depicted solution refers to the same area as the work presented here. However, the system is only relevant in mines with toxic gases, not being useful in surface mines. Also, the presented prototype does not show the final version of wearable in use, a fact that makes harder to estimate its behavior in a real scenario.

## 4. Wecost—Wearable Create OS Tool

This section presents the WeCOST, its architecture, and main functionalities.

### 4.1. Scope of Use

The process to optimize software for embedded systems can become an arduous job, once it may require a whole rework for each device or prototype being designed. This premise is still valid when analyzing wearables using an OS, as specific customizations must be conducted taking into account hardware components, as well as system requirements and functionalities.

As already stated in Section 2.2.3, given the relevant requirements variability and application contexts, building an OS for each wearable solution/prototype becomes a costly task. Sometimes software is no longer prioritized against hardware, often because the latter one presents results in a short time.

The tool presented in this work (WeCOST) has specific applicability in wearable solutions using conventional embedded OS. WeCOST can be taken into account to build wearables classified in any of the layers presented in Section 2.2.5. The great advantage of the tool lies in the use of applicability and requirements specification to decide which software part/module will be improved. This fact allows focusing on specific points of the OS, instead of trying to improve the OS as a whole.

### 4.2. Architecture

**Figure 3:**
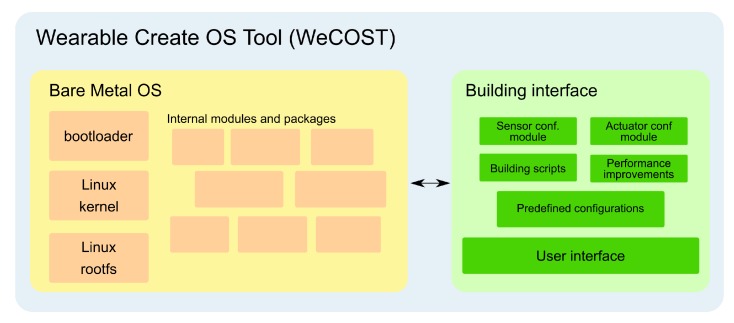
General architecture, main blocks and internal organization of wearable create operating system tool (WeCOST) components.

Figure 3 depicts the WeCOST proposed solution architecture with its two main blocks: “bare metal OS” and “building interface”. Internal communication is handled by the tool, which manages the procedures to generate the OS.

- Bare metal OS: this macroblock specifies how a previously prepared OS is composed. WeCOST uses a base OS as the improvements starting point. This block defines the standard parts of an embedded OS, such as bootloader, kernel, root file system, and other mandatory modules. Until now, WeCOST uses a Linux distribution as the base, since it is popular with a large activity community of collaborators and users. Moreover, Linux already supports many hardware sensors/actuators used in wearables, with device drivers being kept up-to-date. Still, there is also support for compilers, interpreting languages, user-level applications, and so on. This work has taken into account the Buildroot system [108] to craft a reference Linux distribution, used in every OS generated with the tool. The OS kernel and root file system has only the strictly necessary software components (packages and modules) aiming to avoid expending resources with unnecessary parts. If for some reason such default Buildroot configuration does not fill the solution needs, end-user may replace the entire block with a manually customized image; and
- Building interface: composed by a set of configuration files, it provides an interface to the end-user to select desired features of the crafted OS. The system can receive data about functional and non-functional features. For instance, the user can select which sensors/actuators will compose the wearable device, and the tool will attach the necessary software to the OS. Similarly, other system features may also be specified, such as whether it is I/O bound or not, or even if data processing occurs locally or remotely, and so on.
  - Sensor/actuator configuration modules: manage and configure the firmware to support desired sensors and actuators hardware;
  - Building scripts: analyze the end-user choices, and apply the necessary actions to build the OS through the Bare metal OS block;
  - Performance improvements: change the variables values (Section 4.3—non-functional configurations) in order to comply with the focus aimed by the end-user on WeCOST;
  - Predefined configurations: used as a background to construct the new OS. A default Linux distribution was crafted to be used as a reference, with minimum options to boot-up a new wearable OS; and
  - User interface: entry point where the end-user can interact with WeCOST. Selections made here will directly reflect on the OS built with the tool.

### 4.3. Main Functionalities and User Interface

**Figure 4:**
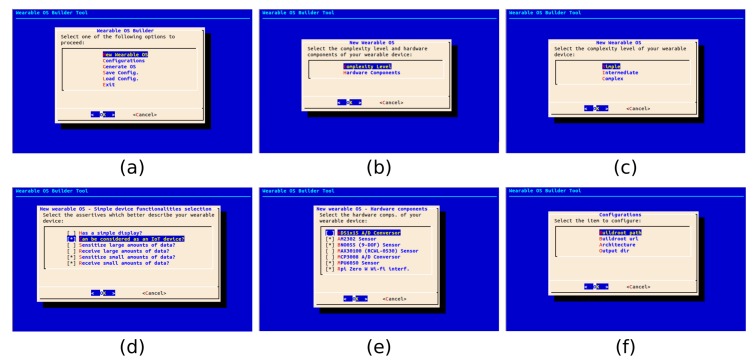
WeCOST screenshots: (**a**) main screen. (**b**) Options selection for “complexity level” and “hardware components”. (**c**) Selection of “complexity level”. (**d**) Example of screen showing assertives of a “simple” wearable device. (**e**) Screen presenting hardware components that can be selected. (**f**) System configurations screen.

WeCOST features belong to two main groups, which consists of a set of configuration variables used to guide the OS development process. From the main menu (Figure 4a), end-user controls any configuration variable related to OS components or improvements.

- OS-related options: allow the end-user to define OS general and specific aspects. This option is subdivided into two other configuration groups.
  - Non-functional configurations: WeCOST provides a way to try to improve OS performance tuning kernel and file-system specific features. In order to map the improvement points and their values, the end-user must inform the tool about the system behavioral characteristics. Based on previous research [31], wearable devices separate into three different categories according to their complexity: Simple, intermediate and complex (Figure 4b,c). By answering questions about device-specific functionalities (Figure 4d), the end-user intrinsically provides input information that supports WeCOST while adjusting and improving the wearable OS performance. Using the answers from the end-user, WeCOST tunes the following variables in the generated Linux OS: I/O caching, I/O scheduler policy, I/O latency time, swap memory sizes, and cache memory policy;
  - Functional configurations: the tool provides a way to choose sensors/actuators set used in the final solution. Once selected, the related hardware will have its specific firmware added to the final OS image (Figure 4e). This functionality aims to ease the task of porting new hardware when creating a wearable prototype or even a product. Moreover, new firmware can be added to WeCOST as soon as new hardware is available to be used in the wearables.
- System-related options: system modularity also allows the end-user to fill out a set of variables describing the path/address of each dependent module (Figure 4f). For instance, a specifically customized Buildroot version can serve as background to start the OS creation process, instead of using the default one. Although this version only covers ARM architecture, WeCOST may, in the future, support different architectures as soon as tool new versions are released;

The WeCOST user interface was created using bash script language as a mean to resemble other previously existent tools. This way, end-users are not required to install any graphical package in order to have the tool working. Options selection is possible using a simple keyboard in a checkbox schema.

### 4.4. Performance Improvement Approach

During user interactions with WeCOST, hardware and functionality choices have a direct relationship with the number of software packages into the final OS version. However, after the initial file system creation, WeCOST performs specific configurations to improve OS performance according to the end-user specification.

Settings configured with WeCOST during Linux-based OS creation focus on the following items and their respective associated files:

- Readahead policy when reading data:
  - /sys/block/sda/queue/read_ahead_kb
- Cache writeback policy when writing data:
  - /proc/sys/vm/dirty_writeback_centisecs
  - /proc/sys/vm/dirty_background_ratio
  - /proc/sys/vm/dirty_ratio
  - /proc/sys/vm/dirty_background_bytes
- Cache writeback threads actuation time interval:
  - /proc/sys/vm/dirty_expire_centisecs
- Swap aggressiveness:
  - /proc/sys/vm/swappiness
- Scheduler policy and latency:
  - /sys/block/sda/queue/iosched/low_latency.
  - /sys/block/sda/queue/scheduler.
  - /sys/block/sda/queue/iosched/quantum.

Values applied to each item above were chosen based on a benchmark specifically developed for wearable computing environment [32] which take into account: I/O operations, processing, graphics, and parallelism. This work prioritizes these operations because they have, in the authors perceived and based on previous work, significant relevance in any wearable device, regardless of whether it is a prototype or a final product.

This research has implemented and executed a particle swarm optimization (PSO) algorithm for each test class. PSO execution takes into account 50 particles and a maximum of 30 iterations. Optimal values found are then used as input in WeCOST, which is in charge of configuring these values in the OS final version according to the decisions made by the end-user.

As an instance, the specific wearable mining prototype proposed in this paper prioritizes I/O operations (since platforms continuously read data from sensors) and processing (since a significant data load is forwarded to the CPU to process information received from sensors). In this case PSO was then executed, taking into account only I/O and processing tests.

## 5. Mining Environment and Test Methodology

This section introduces how the WeCOST tool was validated against a real appliance, showing the applicability environment, the process to design and build a wearable, as well as the settings used to benchmark its performance.

### 5.1. General Context

A surface is a mining category where materials are extracted from the terrain surface, or near it [117]. Minerals can be gathered from the surface or obtained digging vertical benches, which leaves structures that resemble walls in the relief. This activity has a significant impact on the gross domestic product (GDP) of developing countries, being responsible for substantial annual revenues [118,119]. Also referred to as “open-pit” or “open cast mining”, the activity involves specialized structures and machinery, as well as human resources.

Surface mining processes often also involve several challenges, including environment care and safeness protection of persons associated with the process, directly or indirectly. Incidents are likely to become relevant natural and social disasters [120,121,122], in a way that initiatives to reduce or minimize their impact become a relevant topic. For instance, one of these challenges is related to the treatment given to the mining waste, that must be handled in a proper way trying to minimize its further impact.

#### Risks to Workers

In addition to major disasters, surface mining workers are also subject to threats different from those in other types of mines.

- Sun exposure time: the long exposure time to the sun and the inhospitable environment can, in the long run, harm workers health. As an example, dehydration, skin burns, and skin cancer are some consequences of sun exposure long time [123];
- Air quality: surface mining regions commonly have a large number of dust particles suspended in the air. Continued inhalation of these particles can cause respiratory distress or even long-term illness [124]; and
- Others: factors such as proximity to heavy machinery, rugged relief, and others may also create significant hazards to the employees.

This paper shows a surface mining wearable device that continuously monitors workers’ health. Data analysis from specific sensors makes possible to watch previously presented risks.

### 5.2. Wearable Device for Mining Environments

In addition to WeCOST, the authors also propose a novel wearable device for surface mining, intending to empirically validates the software tool. This wearable prototype can be majorly used by people who work in surface mining environments. A set of specific sensors were used to allow employees continuous monitoring when in the field. This solution stands out for the possibility of immediate notification when detecting any change in the monitored variables. This continuous monitoring allows quick feedback of responsible entities, reducing the chances of further complications. Next subsections provide a general view about the main functionalities and the prototype building process.

### 5.3. Prototype Modeling

The wearable device presented in this work follows the concept of sensor nodes. Each hardware in charge of gathering information represents a node. For its turn, these nodes forward the retrieved information to a central CPU. There, data is processed and sent to a central server using a wireless link.

**Figure 5:**
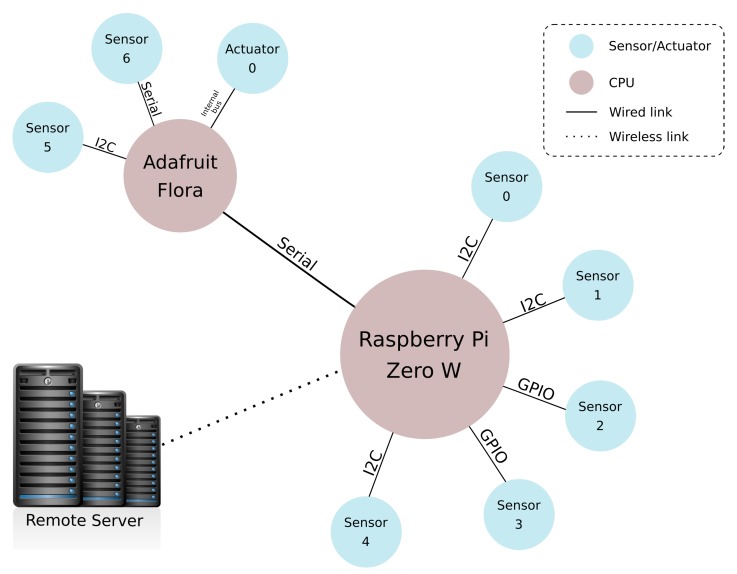
Wearable prototype for surface mining: CPU, sensor nodes and communication links organization.

Figure 5 shows the wearable prototype components organization. The processing load divides into two platforms (CPUs) using different hardware: “Adafruit Flora Playground” and “Raspberry Pi Zero W”. Around these CPUs, there are a set of sensors in charge of collecting information from environment’s and user’s body. Periodically, retrieved data is analyzed in each platform, which, if necessary, sends a notification to a remote server. As pointed out in Figure 5, the Raspberry Pi platform acts as a communication master, controlling the bus when interacting with Adafruit Flora. Components communication are done through specific buses as presented on the picture.

Table 4 presents the set of sensors/actuators attached to each platform. Still, this table also shows what each component monitors and the sampling frequency interval. Indeed, this sampling frequency is intrinsically dependent on functional characteristics. For instance, temperature/humidity data can be retrieved over a frequency greater than information from IMU, since the former one takes longer to change.

**Figure 6:**
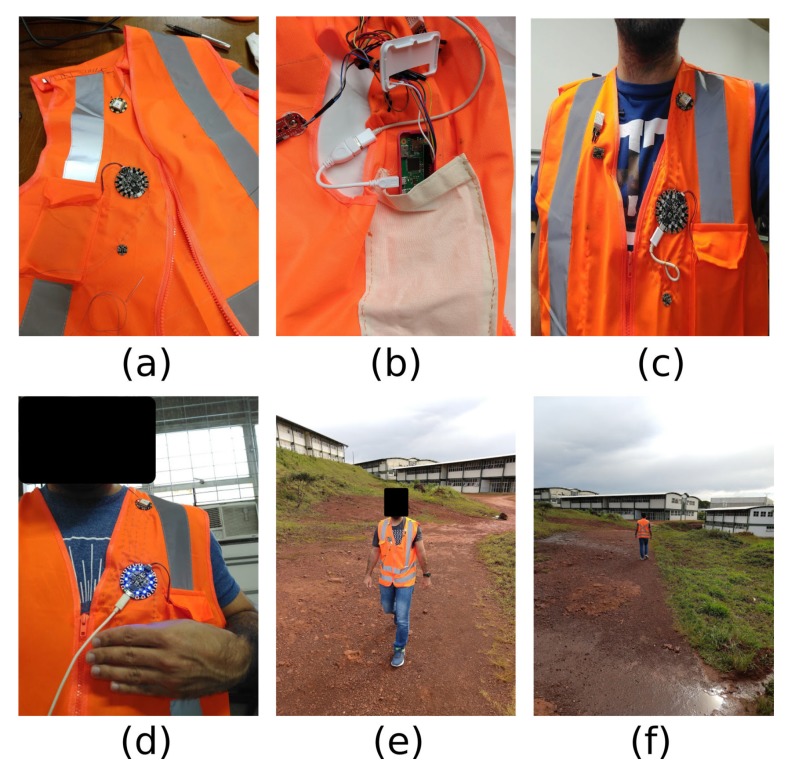
Wearable prototype for surface mining environments: (**a**) prototype during its development. (**b**) Prototype internal pocket holding Raspberry Pi Zero W platform. (**c**,**d**) In-lab development tests. (**e**,**f**) Prototype deployment, field tests and validation.

Figure 6 presents the resulting prototype. Components related to Adafruit Flora are interconnected using conductive threads (Figure 6a), while the remaining sensors connect to the Raspberry Pi Zero W through conventional wires (Figure 6b,c). Raspberry Pi Zero W and power battery are not visible in the images, once their slots are internal (Figure 6b) and external pockets, respectively. Prototype tests and validation took place through two steps: development tests within laboratory (Figure 6c,d) and in-use tests in the field (Figure 6e,f).

#### Main Functionalities

Sensors listed in Section 5.3 have been used to build the wearable prototype features. Each functionality listed below aims to monitor the activities and health condition of a user within the surface mining context.

- Fall detection: the system ran a fall detection algorithm, created through IMU data analysis. Every time the system detected a fall beginning, the platform increased sensor sampling frequency aiming to have access to more information until the fall ends. Once it occurred, the platform sent a notification to the remote server through a wireless link, pointing the GPS coordinates attached to the user location;
- Internal and external temperature: users wearing the developed prototype usually receive a direct incidence of sunlight. Therefore, the solution continuously monitored environment temperature and humidity so that a variation beyond predefined limits generates a notification to the remote server. If necessary, a message can be sent back to the user, reminding him to take some action, such as drink more water;
- Muscular effort monitoring: each user’s muscular effort was monitored and classified in “high effort”, “medium effort” and “low effort” according to the force level detected by EMG sensor. Through the data collected from the left arm, the system can infer the total effort of both arms. If it detects a “high effort”, a notification will also be sent to the remote server. Similarly to temperature/humidity monitoring, the user may be instructed to take a break;
- Blood pressure monitoring: long periods of sunlight exposure and physical effort in the field can also cause changes in blood pressure/oxygen levels. Thus, from time to time the user must check these variables using a specific sensor. Data was classified as “pulse min/max” and “oxygen min/max”, where any value outside these intervals make the system notifies the remote server;
- Visibility increasing: surface mines often use off-road vehicles. For a person, being detected by such vehicles may be important in order to avoid an accident. Especially in periods with reduced natural light, such visibility significantly decreases. Thus, a set of light emission diodes (LEDs) are activated once the wearable luminosity sensor notices a reduction in the environmental light level (Figure 6d).

All the functionalities listed above can contextualize with the user’s physical position through GPS coordinates. If necessary, these coordinates allow a more quick response, once the individual’s location is already known and tracked.

Additionally, a doctor/specialist in the health area must evaluate the data/notifications received in the remote server. The system proposed here does not take decisions by itself, once this is the responsibility of a qualified professional.

### 5.4. Tests Methodology and Performance Measurement

This subsection focus on presenting tests methodology and how to measure performance when comparing WeCOST-generated OS, against a conventional embedded OS without improvements. Tests depicted here were executed in the previously mentioned wearable prototype for surface mining. Two other already existing wearable prototypes were also exclusively used to analyze platforms initialization time.

#### 5.4.1. Performance: Processing and I/O

The performance evaluation examined a set of OS-relevant variables associated with CPU and I/O operations. In this work, the authors understand that these variables best demonstrate the system workload. The following OSs were used for tests execution:

- Not improved OS (NimOS):
  - Common embedded OS for Raspberry Pi Zero W platform, using a Raspbian GNU/Linux 9 (stretch) version [125], kernel 4.4.34+.
  - In addition to the software that comes with this OS, packages to support hardware prototype (Table 4) was also installed.
- Improved OS (ImOS):
  - OS created using WeCOST, which contemplates specific requirements related to the wearable prototype.
  - In addition to the software to support hardware prototype (Table 4), only strictly necessary packages (to boot related platform) were installed by WeCOST when generating this OS.

Ten runs were executed in each case for every scenario. The OS was restarted between two consecutive executions intending to avoid any cached data. Additionally, OS stores performance variables in a log file every two seconds.

#### 5.4.2. Energy Consumption

A specific test was made to measure software impact in wearable energy consumption. The evaluation carried out in this work has examined the already mentioned prototype. Software impact in energy consumption was measured taking into account the two OSs using a specific protocol (Table 5). This protocol separates the analysis according to events occurrence. The wearable prototype was managed to start the events at specific time windows, easing the analysis task.

Energy consumption estimation uses platform current measurements. Values were retrieved using an external Arduino Uno board connected to a current sensor (ACS712 [126]) monitoring the power lines.

#### 5.4.3. Availability

Wearable devices need to be available as soon as the users wear them. This fact implies the necessity of a quick platform initialization in order to avoid data loss. Especially for continuous monitoring applications, data loss can lead to false positives/negatives in some situations.

**Figure 7:**
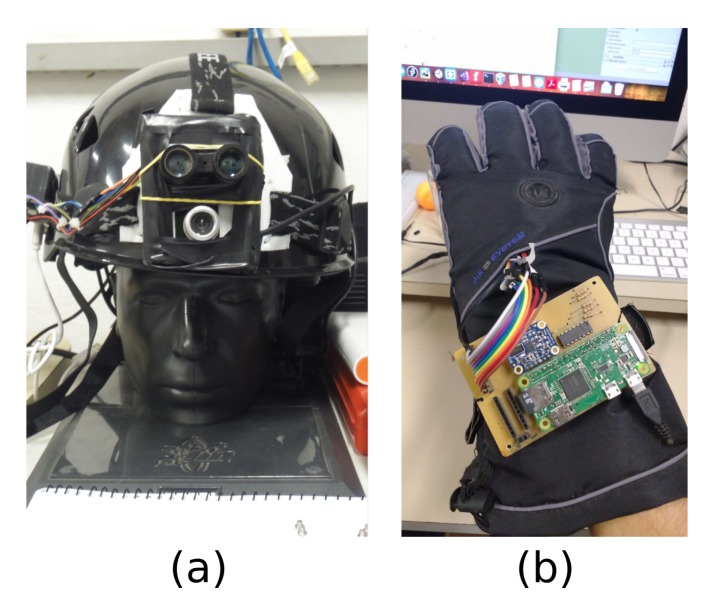
Prototypes exclusively considered during evaluation of platform initialization time: (**a**) wearable helmet prototype. (**b**) Wearable glove prototype.

In this manner, this research has evaluated the requested time to initialize the proposed wearable prototype executing both OSs mentioned in Section 5.4.1. Besides that, as a means to increase evaluation reliability, this work also analyzed the initialization time of two additional already existent in-lab prototypes (Figure 7):

- Wearable glove: allows manipulation of virtual objects with haptic feedback. This solution can be applied to rehabilitation tasks, remote control of physical objects, and others. The prototype is composed of a Raspberry Pi Zero W running a Raspbian Stretch Lite OS with the following sensors/actuators: Flex sensors, BNO055 (9-DOF IMU) and vibra-call motors; and
- Wearable helmet: works as a data gathering tool for ecological studies. Information acquisition process in ecological field research often uses only manual tools [127] and frequently happens in hazardous conditions, such as treetops [128]. The prototype is composed of two different Raspberry Pi Zero W boards running Raspbian Stretch Lite OS with the following sensors/actuators:
  - Board 1: Lidar Lite V1 and BNO055 (9-DOF IMU) sensors; and
  - Board 2: USB camera.

Initialization time considered here includes the interval between power on and platform availability to be used by any user application.

#### 5.4.4. DSP Performance

The significant advantage of using WeCOST is intrinsically related to the wearable application scope, allowing us to delimit software components that will be within the OS. This fact helps to reduce OS size, also diminishing the use of internal resources. As a result, solution functional/non-functional requirements allows making performance improvements at specific points.

However, due to the functional characteristics of particular wearable classes (such as healthcare, sports, individual sensing, and others), it is desirable to evaluate the behavior of an OS when intensively performing DSP-related operations. Thus, in addition to the performance tests described in Section 5.4, the WeCOST tool was also evaluated against a DSP benchmark.

This article has taken into account the liquid-DSP library benchmark [129], which can be taken into account to define the speed each signal processing method/technique can run on every used platform. The following methodology conventions were used during OSs evaluation:

- Test set compares the performance of both OSs (ImOS/NimOS) listed in Section 5.4.1 when using DSP-related operations;
- Confidence level of 95% (with error < 0.1%) was considered;
- Performance evaluation:
  - Each Liquid-DSP benchmark execution runs 785 different tests;
  - This research has conducted 15 executions of liquid-DSP benchmark in every OS (ImOS vs. NimOS);
  - Benchmark covered topics such as filters, fast Fourier transforms (FFT), equalization approaches, checksum methods, modulation/demodulation algorithms, matrix manipulation, and others;
  - The number of executed operations per second (operations/(s)) was used as performance metric. The higher the operations/(s), the better the OS performance.
  - Analysis presented in Section 6.4 was made considering the operations/(s) mean value of 15 executions of each of the 785 tests.

It is also valid to note that the same set of configurations made for the mining prototype were kept in OSs used by these tests. Extended improvements can be further made in DSP-related operations as soon as a specific requirements are defined.

## 6. Results and Discussion

This section presents the tests and main results in the same organization as given in Section 5.4. Except for “availability”, all the results use only the vest prototype for mining environments as the validation platform. At the end of each category, there is a discussion about results, explaining main conclusions and possible hypothesis to obtain them. In general, WeCOST-generated OSs perform better than generic embedded OSs. Results depict a relevant performance variation.

### 6.1. Performance

Processing load (CPU usage) and I/O variables aim to show the behavior of different OSs when subjected to a common wearable solution.

#### 6.1.1. CPU

**Figure 8:**
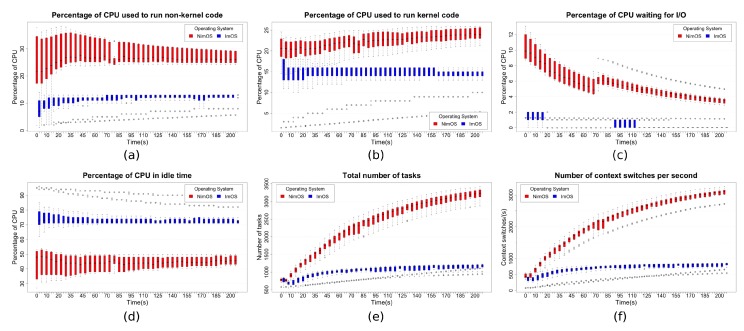
Wearable prototype CPU performance when running not improved operating system (NimOS) and improved operating system (ImOS): (**a**) Percentage of CPU time running non-kernel code. (**b**) Percentage of CPU time executing kernel code. (**c**) Percentage of CPU time waiting for input/output (I/O). (**d**) Percentage of CPU time in idle time. (**e**) Total number of tasks managed by the system. (**f**) Total number of context switches per second.

The CPU usage is a relevant variable in a wearable computing environment. A high or low processing load can demand expensive or cheap hardware. Figure 8 outlines the main results regarding CPU usage variable. Boxplot graphs shown in this figure consider data mean values collected through the ten executions in an interval of 3:25 min. In this manner, each box represents data agglutination of all the runs in that seconds of the time slot.

Figure 8a shows a consistent improvement when using the OS generated with WeCOST. During all the sampled time, ImOS has a better performance than NimOS. A higher variation in CPU time occurred during the initial seconds of application execution. However, after a while, the difference between both OSs, while CPU runs non-kernel code, was stabilized around 15%.

Figure 8b corroborates results presented above. Again, ImOS has a better performance than NimOS. After some initial variation, the difference between both OSs stabilizes around 7%. It is valid to highlight that in ImOS, more standardized measures can be seen, especially after the initial part of application execution. Improvements made on ImOS can explain this behavior. Removal of unnecessary components allowed the ImOS to have a lighter system-level workload, without having to change application-level solution. This fact reduced the CPU percentage while processing kernel code.

Figure 8c presents other relevant data, which shows the CPU percentage time waiting for an I/O operation. The higher the values presented in this figure, the greater the number of I/O operations. Thus, data related to NimOS presents a decreasing behavior since the application was started. This same pattern can be seen right after the 70th second of the time window. The hypothesis considered here is that NimOS is suffering from I/O operations of some system component(s) outside the considered wearable application. Generally, ImOS CPU time “waiting for I/O” is lower than NimOS, sometimes six times lower.

Complementing the previous results, the data presented in Figure 8d shows that CPU running ImOS stays most of the time in idle. Such data makes sense when compared to previous results, once the solution running NimOS frequently spends more time executing other codes (kernel and non-kernel ones). This fact also allows concluding that, in both cases (especially in the former one), the hardware platform has more resources than needed in the proposed wearable prototype. In a final product, a hardware specification might be simplified, reducing the costs.

Additionally, the results from Figure 8e,f can be analyzed together. Figure 8e presents the total number of tasks running in the entire wearable platform. In NimOS, the tasks number tends to increase over time, as other system components keep creating them until it stabilizes, although the graph does not present this stabilization point. The reason behind such behavior requires a more thorough investigation since it is necessary to screen NimOS tasks execution. Conversely, because of prototype application initialization, the tasks number in ImOS also increases during the first execution seconds. However, this behavior happens smoothly. After around three minutes of execution, the tasks number in NimOS was almost three times bigger than in ImOS. Figure 8f repeats this general behavior. The context switches number reflects the system tasks number: The more tasks the OS creates, the more context switches CPU performs to execute all of them. Results present almost the same curves when comparing NimOS vs. ImOS, with significant improvements in ImOS.

In an overview, it is possible to figure out that the variation presented here, between ImOS and NimOS, relates intrinsically to the improvements made with WeCOST. Adding only required components makes the system lighter. Still, configurations made in the scheduler, cache policies (readahead/writeback), and system threads, also have an impact, since CPU interventions in the system tasks may be required less frequently. Briefly, processing is no longer spent on unnecessary software parts, allowing the hardware to dedicate to the truly needed tasks. Still, this fact also allows the use of less powerful hardware, which tends to have lower costs.

#### 6.1.2. I/O

**Figure 9:**
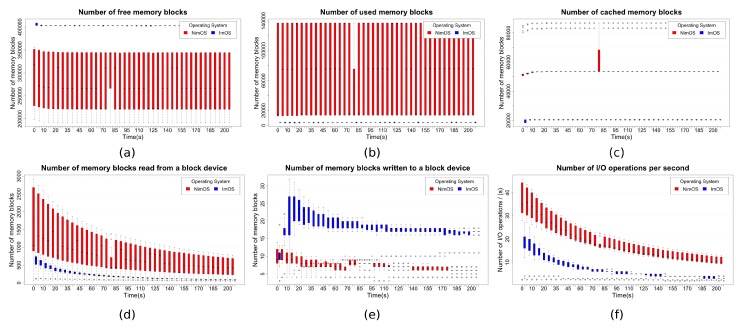
Wearable prototype I/O performance when running ImOS and NimOS: (**a**) number of free memory blocks. (**b**) Number of memory blocks in use. (**c**) Number of cached memory blocks. (**d**) Number of memory blocks read from a block device. (**e**) Number of memory blocks written to a block device. (**f**) Number of I/O operations per second.

The I/O operations number can influence the whole system performance. In wearable devices context, use of unnecessary software (or even hardware) may impose I/O operations that also affect system resources. Figure 9 presents the main results regarding I/O-related variables and their usage. Boxplot graphs consider data mean values collected through the ten executions in an interval of 3:25 min. Each box represents data agglutination of all the runs in that latest time slot.

Figure 9a,b presents complementary results and shows memory blocks usage in the proposed wearable running ImOS and NimOS. There are a significant number of free memory blocks in ImOS, while this number varies significantly in NimOS (Figure 9a). In contrast, the amount of used memory is low in ImOS and high in NimOS (Figure 9b). Additionally, these results also show that the proposed prototype requires a minimum usage of main memory, once it is possible to run it using ImOS. However, as can be seen in NimOS data, a not improved OS can demand a high amount of memory, generating a significant discrepancy. Indeed, a more detailed analysis allows observing a relevant variation in the number of memory blocks used in NimOS. This fact means that additional resources are employed to allocate/free the memory blocks by the system.

Figure 9c provides another memory analysis. This graph shows results regarding cached memory usage by the system, while wearable application runs. Again, there is a relevant difference between ImOS and NimOS, with the former one using more than twice fewer memory blocks of cache. A curious event happened during 1:20 min of analysis, with the number of cached memory blocks varied significantly in the solution using NimOS. As this same event did not occur in ImOS execution, the hypothesis to this variation can relate to some OS event.

Figure 9d,e shows the behavior of the analyzed systems regarding amount of read/written data from/to a block device. A GNU Linux block device usually is associated with physical disks. However, especially in the embedded systems context, the same concept also applies to solid-state memories. The platform employed in this work uses a Raspberry Pi Zero W board, which also maps main (ramdisk) and secondary (flash) memories as block devices. Additionally, the wearable sensors using Inter-IC Communications (I2C) work as a block device, since they trust in system management bus (SMBus) [130] protocol. When analyzing the number of memory blocks (Figure 9d), it is possible to verify a disparity between ImOS vs. NimOS. The number of memory blocks reads was commonly greater in NimOS than in ImOS. A significant variation happened at the beginning of the application execution. This behavior matched the expectations since NimOS has a greater number of software components using available block devices. In contrast, Figure 9e shows an unexpected behavior. Although it is not very significant, the number of memory blocks written when using ImOS exceeds the corresponding value when using NimOS. The same type of variation occurred during the first minute of execution, and after that values become almost stabilized, with little changes. A valid hypothesis to explain this data resides in cache memory policies and scheduling, once WeCOST tunes these variables in ImOS according to the type of wearable application. This fact, allied to a reduced number of memory blocks reads, may generate a room to expand the memory block write operations number.

Finally, Figure 9f outlines the number of I/O operations per second each OS performs. This graph can be interpreted as a kind of data summarization since it reflects all the operations previously mentioned in other graphs in Figure 9. Curves presented in this graph show a better ImOS performance in comparison to NimOS. Even the same initial variation can also be seen at the beginning of this figure, including the behavior already presented in other results. I/O operations per second in ImOS can even be, in some situations, twice lower than in NimOS, demonstrating improvements impact in OS generated with WeCOST.

An overview of I/O results shows the impact generated with ImOS improvements. In almost every scenario, ImOS performance surpasses NimOS. In addition to specific changes, merely reducing the unnecessary software components enhances overall performance. Besides that, the results from this section support those previously presented.

### 6.2. Energy Consumption

**Figure 10:**
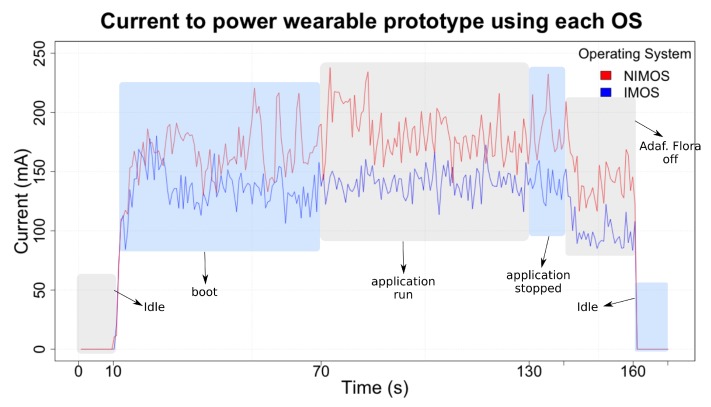
Wearable vest prototype demanded current (mA) when running application in ImOS and NimOS.

Energy consumption evaluation aims to provide a set of discrete values describing energy impact in a wearable solution. This variable has a direct influence on wearable devices autonomy, possibly affecting consumers acceptance level. Figure 10 presents results regarding the device’s required current to power the whole solution. As the test protocol (Table 5) previously outlines, the analysis has proceeded involving different phases of system execution.

In a general analysis, improvements conducted in ImOS also reflected in lower energy demand. During the initial and final 10 s, the system was off and demanded no current. In the next 60 s, platform initialization occurred, showing significant variation in the current values. This stage also shows that sometimes ImOS current values are higher than those presented in NimOS, although in a short period.

The next part comprises data from current when running the wearable application. With some variations, the same behavior occurred in this third part. Wearable solution running in ImOS performed better than in NimOS. In the beginning, it was possible to observe a peak in NimOS current consumption. Authors infer that some system event caused that current increase, once it cannot be seen in ImOS.

The same patterns appeared in the “application stopped” stage. These results suggest wearable application having little influence on the platform energy consumption. It is mainly because the hardware still required current to keep them powered.

The “Adaf. Flora off” part displayed a relevant difference. This result reflects the amount of current required to power Adafruit Flora and its associated sensors. In both scenarios (NimOS vs. ImOS), it is possible to verify a reduction in required current. Additionally, as in the previous stages, the solution running ImOS still performed better than NimOS.

An overview analysis shows ImOS performing better than NimOS. As in the previous evaluations, the explanation regarding this behavior commonly resides in the software components loaded into the OS. Here, this same interpretation is possible. Table 6 shows consolidated data regarding Figure 10, where it is possible to verify a sensible increase in the current required by NimOS, which carries more software modules/components than ImOS. Furthermore, in a stage-by-stage analysis, the current variation presented in NimOS is also higher than in ImOS.

Table 7 displays a rough long-term estimation regarding energy consumption. Only the “application run” stage was taken into account in this analysis, as the remaining parts just run during a short period at the beginning/end. Data presented in this table allows verifying the impact an improved OS has in a long-term execution. Hardware platform running ImOS performed almost 30% more efficiently than NimOS. When considering a power source of 12,000 mA, this fact can be translated into an increased autonomy, since the ImOS platform can stay running for more than 7.0 days. In contrast, in this estimation, a platform with NimOS cannot surpass 5.5 days.

### 6.3. Availability

**Figure 11:**
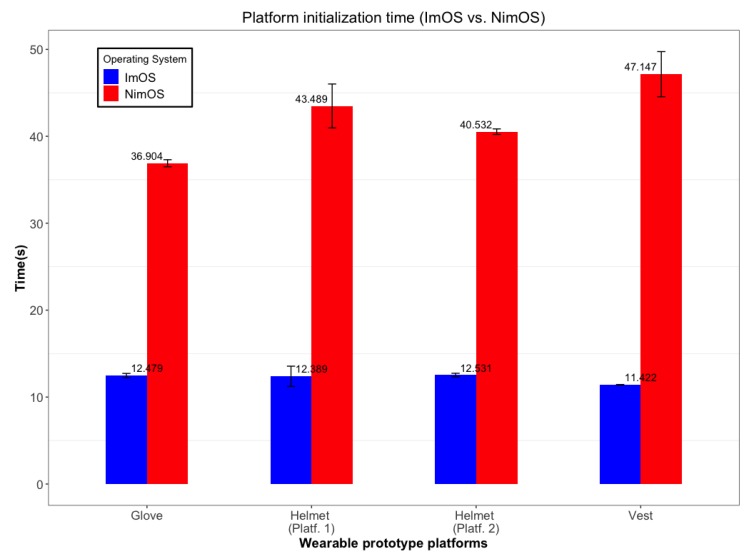
ImOS vs. NimOS: initialization time of all wearable prototypes.

Results presented here aim to provide an overview regarding improvements impact of WeCOST in the OSs. Data shown here helps to perceive the initialization time importance on a wearable platform. As soon as the platform initializes, the final application can also load, starting to process data collected with the sensors. If the platform booting process takes a long time to complete, there is a risk of user data loss. Especially in wearable applications with bio-signals analysis, this fact may have a significant impact on the whole solution. Results presented in Figure 11 summarize the time required to boot each wearable prototype analyzed in this work. In each of these prototypes, ImOS and NimOS represents a way to infer the improvements magnitude.

The platforms used in this test presented similar results. NimOS takes a significant time to boot any of the considered wearable setups. In all the results, ImOS initializes at least three times faster than NimOS. In an extreme case (wearable vest for mining environments), ImOS is 4.13 times faster than NimOS. Another unique result took place in the Helmet prototype. Since it used two boards with different applications, it was scrutinized in an isolated way. However, the behavior was almost the same, with NimOS requesting a significant time to bring OS up. Still, it is valid to highlight that the OS reached this boot time without any initialization specific improvement. Results listed here reflect only improvements made in wearable OS parts other than initialization, in a way that these values can be further improved if the tool enhances OS parts specifically related to boot time.

In general, the results presented in this section corroborates the previous ones. ImOS requests less time to initialize all the OS software components. Another point to consider is the variation presented in the above results. Variations in the ImOS boot times were always significantly smaller when compared to the NimOS versions. These results suggest that in addition to being reduced, boot times have become more constant. Such behavior is highly desirable for the wearables since it increases system predictability as a whole. A future effort can be made in WeCOST solution to improve boot time, further enhancing results outlined here.

### 6.4. DSP

Digital signal processing is commonly used by several categories of wearable devices, especially those associated with healthcare environment [98,99,100]. Improving DSP operations performance may allow the whole system run more efficiently. Such improvements can avoid, in a short time, the need to change hardware components.

DSP performance results presented in this Subsection considers the methodology described in Section 5.4.4. Results were classified according to the classes defined in Table 8. Each class encloses how better an OS performed when compared to the other one. In this way, a more efficient OS should perform as many operations as possible in a given time. Results shown below, provided by the benchmark, consider as an evaluation metric the number of operations performed per second (operations/(s)).

**Figure 12:**
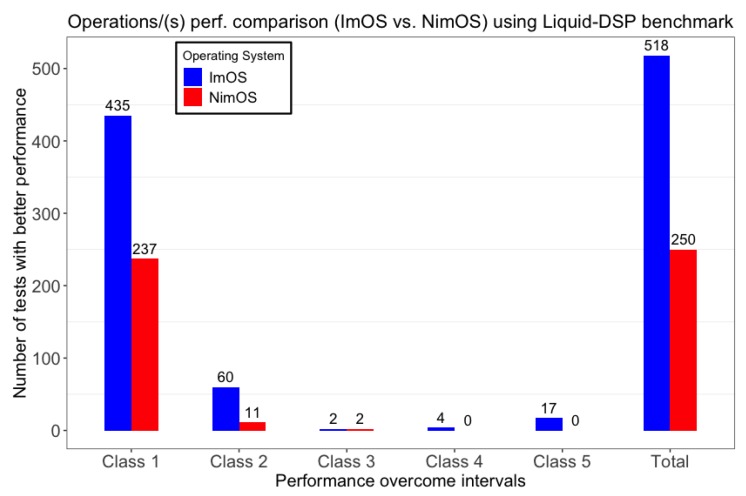
ImOS vs. NimOS: Number of tests in which an OS overcame the other one. Classes defined in Table 8 identify improvement percentage obtained by the faster OS.

Figure 12 shows the number of tests in which ImOS has surpassed NimOS performance (or vice-versa). Associated classes denote the improvement percentage reached by the faster OS.

Class 1 depicts ImOS surpassing NimOS performance in 435 tests by up to 10% in operations/(s). Conversely, NimOS reached a better performance in 237 tests. In an in-depth verification, the authors found that NimOS best results were related to tests handling data modulation/demodulation. Further investigation is then required to clarify the reasons for such behavior.

Class 2 data shows that in 60 tests ImOS performance was between 10% and 30% better than NimOS. By its turn, the 11 tests where NimOS has surpassed ImOS are all related to checksum (CRC) checking, motivating further investigations and improvements on this part.

Class 3 presents ImOS with better performance (between 30% and 50%) in two tests. Analogously, NimOS also obtained a better result than ImOS in the other two occasions. The tests, where NimOS performed better, are all related to data synchronization when using 16/32 bits vectors as input. Given the number of executed runs and confidence level, a more extensive analysis is also required to understand ImOS poor performance on these two tests.

Classes 4 and 5 enclose four and 17 tests, respectively, where ImOS performance was better than NiMOS. Although the reduced number of tests in these classes, they represent a consistent improvement when comparing ImOS against NimOS. Class 4 results are related to tests with the number of operations/(s) between 50% and 100% bigger in ImOS. By its turn, class 5 is associated to tests where ImOS surpassed NimOS in at least 100% of operations/(s), i.e., ImOS executed at least 2x more operations/(s) than NimOS in the same period.

Despite NimOS still performing better in a certain number of situations, it is possible to observe that improvements made in ImOS had a relevant impact. The tests in which no improvement was noticed are those presenting a smaller performance difference when comparing the two OSs.

In an overview, the WeCOST tool use can be justified when analyzing global results. ImOS reached a better performance in ∼66% of the tests. Analogously, NiMOS performed better than ImOS in ∼31.85% of the tests. The remaining ∼2.15% are tests where ImOS and NimOS reached the same values for operations/(s). Additionally, tests with the highest improvements percentages could be observed when running the tests in ImOS (classes 4 and 5).

Results presented in this subsection can even be further improved when the specification of the final applicability exists. With the definition of functional/non-functional requirements, a more focused set of enhancements can be applied, extending the improvements impact.

## 7. Conclusions

Recent advances in hardware have allowed new and more end-users attractive wearable solutions. From simple to sophisticated devices, wearables have become increasingly ubiquitous and unobtrusive. Currently, solution performance enhancement commonly passes through changes in hardware. These facts force devices into a short life cycle, since a few months later the product may be replaced by its subsequent version.

Improvements in wearable solutions are commonly conducted through hardware changes, although their costs. For complex devices that make use of OSs, changes may require a consistent development effort and time. This reason commonly demands solutions to adapt and reuse smartphones and tablets platforms OSs. Despite its practicality, such solution commonly delivers lower-than-expected performance, forcing the hardware to be more robust and powerful.

In this work, the authors presented a tool (WeCOST) to design and build wearable-specific OSs. WeCOST aims to speed-up the wearable software development process, besides providing a performance-improved OS. Additionally, it was shown how OS improvements might impact in whole system energy consumption, possibly affecting the system autonomy in a long-term.

A novel wearable prototype applied to mining environments was also depicted in this research, as a means to validate the proposed tool and analyze OS impact in wearables. Results compared a not improved OS (NimOS) with the improved one (ImOS), generated with WeCOST. Through the resulting data, it was possible to see the impact a not-improved OS has in general performance. General processing, I/O, initialization time and even energy consumption were affected by OS improvements. Besides development time reduction, WeCOST also contributes requiring less powerful hardware, since resources are better utilized. For instance, results of this work present a significant portion of time where the CPU is idle (around 70%), allowing authors to wonder if a more straightforward hardware platform will fulfil wearable solution needs. Additionally, WeCOST-generated OS has performed well even when evaluated in already existent wearable prototypes. In the end, ImOS was evaluated against NimOS using a specific benchmark for DSP applications. As results reported, in ∼66% of tests, ImOS has performed better than NimOS.

### Limitations and Further Research

The WeCOST-created OS presented in this work supports a specific subset of sensors and actuators. In order to make the tool even more comprehensive, support for new hardware must be included in the next development steps. In fact, because of its modularity, more improvements can be inserted into WeCOST, such as initialization, graphical modules and so on, aiming to use the tool in a large variety of wearables.

## Figures and Tables

**Table 1:** Common sensors used by wearables.

Sensor	Description
**Accelerometer**	Provides the acceleration values in *x*, *y* and *z* axis [39].
**Camera**	Collect images and videos [40].
**Capacitive**	Provides information about variations in electric potential or electric field [41].
**Electrocardiogram (ECG)**	Information about heart activity obtained using electrodes [42].
**Electroencephalography (EEG)**	Information about electrical activity of the brain [43].
**Electromyogram (EMG)**	Data of electrical activity produced by skeletal muscles [43].
**Gas**	Provides information about concentration of specific gases inside an environment (or open areas) [25].
**Global positioning system (GPS)**	Provides global position besides a set of other relevant information [44].
**Gyroscope**	Information regarding orientation and angular speed [45].
**Magnetic**	Data of magnetic fields values [46].
**Microphone**	Sensory and capture sounds [47].
**Oximeter**	Provides information about person’s oxygen saturation [48].
**Photoplethysmogram (PPG)**	Information about changes in blood volume of vascular part of tissues [42].
**Pressure**	Measures the force amount applied to a surface or object [49].
**Strain**	Provides data regarding the strain of a surface or object [50].
**Temperature**	Information about hotness/coolness of an environment/object [51].

**Table 2:** Common applicability areas where wearables are used.

Area	Description
**3D modeling**	Devices used to model 3D representations of the real world [52].
**Agriculture**	Solutions to support development of agriculture tasks [53].
**Artistic**	Wearables used in artistic performances [54].
**Civil construction**	Devices that assist in light and heavy tasks in civil construction [55].
**Communication**	Wearables to ease or increase communication between two or more people [56].
**Cooking**	Solutions to assist in the tasks within a kitchen, helping users to cook [57].
**Driving**	Equipment aiming to assist drivers to increase their safety and focus while driving [58].
**Education**	Devices used in classrooms to support teachers and educators [59].
**Entertainment**	Solutions used with entertainment purposes [60].
**Envir./objects/individual sens.**	Wearables used to sense information from users’ context [61].
**Fabric**	Solutions that support the development of new wearable fabrics [62].
**Healthcare/ Medicine**	Wearables used sense users’ bio-signal information [63].
**Maintenance**	Devices that assist users during maintenance tasks [64].
**Military**	Equipment aiming to improve military tasks in battlefield or not [65].
**Navigation**	Wearables used during navigation in indoor or outdoor environments [66].
**Security/Rescue**	Devices covering activities developed during security or rescue operations [67].
**Sports**	Solutions used within the context of sports aiming to improve athletes performance through real-time data analysis [68].
**Textile**	Devices focusing on textiles that may, or may not, be used in more complex solutions [69].
**Others**	In addition to the classes provided above, there still a set of remaining wearables in other applicability areas, such as: geology, mining and electronic devices interaction [25,70].

**Table 3:** Common topics addressed by wearable devices research and development.

Research Topic	Description
**Autism therapy/support**	Devices which provides support or better alternatives to therapy of autism [71].
**Brain-related diseases**	Wearables employed in the treatment of these diseases [72].
**Breath analysis**	Solutions aiming to analyze and process breath pattern aiming to identify specific activities being performed by the end-user [73].
**Cognitive assistance**	Wearables created to assist end-users during basic daily activities [74].
**Context awareness**	Ubiquitous devices that provide context-aware information to the end-users [56].
**Daily life monitoring**	Wearables used to monitor daily life activities, which provides support and contextualized information [75].
**Dementia support/monitoring/ therapy**	Devices applied in dementia support and therapy. Solutions in this class can also be used to monitor end-users in order to improve end-users’ life quality [76].
**General diseases detection**	Devices used to detect any type of disease [77].
**Energy efficiency**	Wearables focusing on energy efficiency improvement. Energy harvesting, energy expenditure reduction and any other technique can be listed in this topic [78].
**Eye tracking**	Solutions aiming to precisely identify the eye, tracking its position and movements [79].
**Fall detection**	Wearables focusing on fall detection and identification of its dangerousness [80].
**Gait assistance/support/tracking**	Devices aiming to provide gait support, tracking or any other assistance [81].
**Gaze tracking**	Devices focusing on eye tracking for gaze direction identification [82].
**Human activity recognition/activities of daily living**	Wearables applied on activities recognition processing information retrieved from IMU sensors. Users’ daily behavior can be mapped through the activities identification [83].
**Indoor/outdoor localization or navigation**	Solutions used to address the problem of indoor/outdoor localization or navigation [84].
**Personal energy expenditure**	Wearables used to gather and process users’ information regarding daily energy expenditure. This problem is commonly associated with healthcare applications to estimate the calories expended during a day [85].
**Physiological parameters analysis**	Devices used to retrieve and process physiological data aiming to infer end-users’ health [86].
**Posture and gesture recognition**	Solutions used to process data from sensors to estimate the posture/gestures made by the user. Such type of estimation can also helps identifying activities performed by the user during a day [87].
**Privacy**	Wearables focusing on users’ privacy increasing [46].
**Rehabilitation**	Devices designed to provide end-users’ support to any rehabilitation approach [88].
**Remote monitoring**	Solutions aiming to provide remote monitoring to the users’ activities. Devices within this class can be used within several different contexts, such as sports, healthcare, security, and so on [89].
**Sleep staging/monitoring**	Wearables designed to monitor users’ behaviour during sleep [90].
**Stress management/monitoring**	Devices designed to monitor and manage users’ stress [91].
**User interfaces/user experience**	Wearables focused on improving users’ interaction with the devices [92].
**Visually impaired people support/mobility**	Devices designed to support and improve visually impaired. users’ mobility [93]
**Others**	Besides the topics previously listed, there exist other less-popular problems addressed by the research. Anorexia therapy [94], color vision impairment [95], general data harvesting [51], drug usage detection/monitoring [96], and others could be added to this list.

**Table 4:** List of platforms (CPUs), sensors and sampling frequency used in the wearable prototype for mining environments.

CPU	Sensor/Actuator	Description	Sampling Freq. (s)
**Adafruit Flora**	GPS	User’s position.	60
	Luminosity	Light luminosity level.	60
**Rasp. Pi Zero W**	Temp./humidity	Sensors collecting temp./humid. from user’s body and environment.	180
	IMU	Data from user’s body acceleration and rotational motion in *x*, *y* and *z* axis.	0.2
	EMG	Information from muscular efforts made by the user.	0.2
	ECG	Sensor retrieving data from blood pressure and oxygen saturation.	3600

**Table 5:** Test protocol used to measure energy consumption on wearable prototype.

Time Interval	Description	Duration(s)
0–9 s	Idle time—waiting time with platform turned-off.	10 s
10–69 s	Boot time—time slot reserved to boot the platform.	60 s
70–139 s	Application run—execution of prototype application.	60 s
140–159 s	Adaf.Flora off—Adaf. Flora turned-off aiming to measure its energy impact.	20 s
160–169 s	Idle time—waiting time with platform turned-off.	10 s

**Table 6:** Improved operating system (ImOS) vs. not improved operating system (NimOS): current (mAh) measurement overview.

Operating	Boot		App. Running		App. Stopped		Adaf. Flora off		Total	
System	Mean	std.dev.	Mean	std.dev.	Mean	std.dev.	Mean	std.dev.	Mean	std.dev.
**ImOS**	132.34	25.73	140.77	10.98	139.70	11.43	100.33	15.88	131.43	22.83
**NimOS**	161.65	32.84	182.43	20.15	185.01	19.60	145.55	20.42	169.37	29.21

**Table 7:** Long-term energy consumption estimation.

Operating	Per Hour (mAh)		Per Day (mA)	Est. Autonomy (days)	Improvement Rate
System	Mean	std.dev.	Total	12,000 mA Battery	NimOS/ImOS
**ImOS**	140.77	10.98	1689.24	7.10	1.30
**NimOS**	182.43	20.14	2189.16	5.48	

**Table 8:** Digital signal processing (DSP) benchmark performance results classification.

Class Name	Interval	Description
**Class 1**	x < 10%	Faster OS executed less than 10% (1.1) more operations/(s) than slower OS.
**Class 2**	10% <= x < 30%	Faster OS executed between 10% (1.1) and 30% (1.3) more operations/(s) than slower OS.
**Class 3**	30% <= x < 50%	Faster OS executed between 30% (1.3) and 50% (1.5) more operations/(s) than slower OS.
**Class 4**	50% <= x < 100%	Faster OS executed between 50% (1.5) and 100% (2.0) more operations/(s) than slower OS.
**Class 5**	x >= 100%	Faster OS executed more than 100% (2.0) operations/(s) than slower OS.

## Abbreviations

The following abbreviations are used in this manuscript:

OSs	Operating systems
WeCOST	Wearable create OS tool
IoT	Internet of things
AR	Augmented reality
VR	Virtual reality
AI	Artificial intelligence
WBAN	Wearable body area network
NFC	Near field communication
GPU	Graphics processing unit
PSO	Particle swarm optimization
IMU	Inertial measurement unit
SoC	System on chip
HMD	Head-mounted display
I/O	Input/output
RTOS	Real-time operating system
CPU	Central processing unit
DSP	Digital signal processing
GDP	Gross domestic product
EMG	Electromyogram
ECG	Electrocardiogram
EEG	Electroencephalography
GPS	Global positioning system
PPG	Photoplethysmogram
UV	Ultraviolet
LED	Light emission diode
NimOS	Not improved operating system
ImOS	Improved operating system
I2C	Inter-IC communications
SMBus	System management bus
DOF	Degree Of freedom
FFT	Fast Fourier transforms
LCD	Liquid Crystal Display
RFID	Radio-Frequency Identification
